# Social learning from humans or conspecifics: differences and similarities between wolves and dogs

**DOI:** 10.3389/fpsyg.2013.00868

**Published:** 2013-12-03

**Authors:** Friederike Range, Zsófia Virányi

**Affiliations:** ^1^Messerli Research Institute, University of Veterinary Medicine Vienna, Medical University of Vienna and University of Vienna Vienna, Austria; ^2^Wolf Science Center Ernstbrunn, Austria

**Keywords:** domestication, local enhancement, human demonstrator, conspecific demonstrator, dog, wolf

## Abstract

Most domestication hypotheses propose that dogs have been selected for enhanced communication and interactions with humans, including learning socially from human demonstrators. However, to what extent these skills are newly derived and to what extent they originate from wolf–wolf interactions is unclear. In order to test for the possible origins of dog social cognition, we need to compare the interactions of wolves and dogs with humans and with conspecifics. Here, we tested identically raised and kept juvenile wolves and dogs in a social learning task with human and conspecific demonstrators. Using a local enhancement task, we found that both wolves and dogs benefitted from a demonstration independent of the demonstrator species in comparison to a control, no demonstration condition. Interestingly, if the demonstrator only pretended to hide food at the target location, wolves and dogs reacted differently: while dogs differentiated between this without-food and with-food demonstration independent of the demonstrator species, wolves only did so in case of human demonstrators. We attribute this finding to wolves being more attentive toward behavioral details of the conspecific models than the dogs: although the demonstrator dogs were trained to execute the demonstration, they disliked the food reward, which might have decreased the interest of the wolves in finding the food reward. Overall, these results suggest that dogs but also wolves can use information provided by both human and conspecific demonstrators in a local enhancement task. Therefore we suggest that a more fine-scale analysis of dog and wolf social learning is needed to determine the effects of domestication.

## INTRODUCTION

Intense research in the last few decades has revealed dogs’ exciting communicative and cooperative skills with humans (Naderi et al., 2001; Szetei et al., 2003; Viranyi et al., 2004; Schwab and Huber, 2006; Kaminski et al., 2012). These abilities are often assumed to originate partly from the dogs’ evolutionary adaptation to the human environment (Hare et al., 2002; Miklosi et al., 2003; Hare and Tomasello, 2005) and partly from their life-long experiences with humans (Udell et al., 2010; Miklosi and Topal, 2011). Up to now, most of this reasoning has been based on the performance of canines in interactions with humans (Miklósi and Topál, 2013), and it has rarely been discussed to what extent the human-like characteristics of dogs originate from the socio-cognitive skills of wolves used in within-species contexts. The emotional reactivity hypothesis is an exception because it postulates that dogs were selected for a decreased level of human-directed fear and aggression, leading to a greater acceptance of humans as social partners, which in turn allows dogs to use intraspecific social cognitive skills with humans (Hare and Tomasello, 2005). According to this, dogs should do better than wolves when humans are involved in cooperative interactions with them but dogs and wolves should show similar cognitive skills when interacting with conspecifics (Hare et al., 2002; Miklosi et al., 2003; Hare and Tomasello, 2005). In order to dissect domestication-caused changes in the social cognitive abilities of dogs, we need to test dogs not only in interactions with humans but in other contexts as well. Furthermore, to learn to what extent dogs’ social cognition (and not only their readiness to communicate and cooperate with humans) differs from that of wolves, we also need to compare dogs and wolves in cognitive tasks with conspecifics. Accordingly, we set out to compare wolves and dogs that were socialized with humans, conspecifics and several pet dogs to a similar extent, in a social learning task using both human and dog demonstrators.

Social learning occurs when an individual’s learning is influenced by observation of, or interaction with, another animal or its products (e.g., Heyes, 1994). For example, animals may learn about differences between foraging patch quality (Valone, 1989), a process dubbed “area copying,” by paying attention to a place or location where a conspecific is showing a species-specific behavior (Giraldeau, 1997). The underlying mechanism, called local enhancement, is mainly perceptual or attentional in nature (Thorpe, 1956) and does not require learning about new behaviors (imitation), object properties (affordance learning) or the goals of the demonstrator (Whiten and Ham, 1992; Zentall, 2004). From the functional point of view, it offers a plausible explanation as to how animals might learn where to find food from others (Galef and Giraldeau, 2001).

To date, while several studies have shown that dogs’ performance can be enhanced by the demonstration of a human (Pongracz et al., 2001) or conspecific model in various experimental paradigms (Slabbert and Rasa, 1997; Pongracz et al., 2004; Range et al., 2007, 2009; Kubinyi et al., 2009), the evolutionary and developmental origins of this performance have not been explored. Reports from the wild suggest that wolves raid the caches of pack members (Dave Mech, personal communication), suggesting that they pay close attention to the location where the pack members hide their food and that they can use that information to find the cache. Alternatively, however, it is possible that they mainly rely on their sense of smell to find hidden food.

In the current experiment, we set out to investigate whether wolves and dogs, raised with the same intensity of human and conspecific interactions, differ in their ability to use a conspecific (familiar dog) and a human as an “informer” in a local enhancement task. On top of investigating if the animals could benefit from a demonstration to find a hidden piece of food in a meadow, we were also interested in whether they would be able to recognize if the demonstrator (human or dog) actually hid a piece of food or only pretended to do so. The latter condition was carried out to test if the animals paid attention to the details of the demonstration. Finally, we retested the animals three times between 3 and 8 months of age to detect possible developmental changes in the cognitive skills of wolves and dogs.

Along the line of the emotional reactivity hypothesis outlined above, we predicted that while both wolves and dogs would benefit from the demonstration of a conspecific, dogs would also be able to use the human demonstration to find the hidden food, whereas wolves would not or at least would do so to a lesser extent (e.g., they would not distinguish between “pretend” and “real” demonstration because they would pay less attention to the details of the human demonstration).

## MATERIALS AND METHODS

No special permission for use of animals (wolves and dogs) in such socio-cognitive studies is required in Austria. The relevant committees that allow research to run without special permissions regarding animals are: Tierversuchskommission am Bundesministerium für Wissenschaft und Forschung (Austria).

### SUBJECTS

All wolves (*n* = 11) that participated in this study originated from North America but were born in captivity. The dogs (*n* = 14) were mongrels born in animal shelters in Hungary. For relatedness, date and place of birth please refer to **Table 1**. All of the animals were hand-raised in peer groups after being separated from their mothers in the first 10 days after birth. They were bottle-fed and later hand-fed by humans and had continuous access to humans the first 4 months of their life. After spending the first weeks of their lives mainly indoors, at the age of 10 weeks the dogs and wolves were transferred to a 400 m^2^ outside enclosure with access to an indoor room (puppy room), where the hand-raisers, one at a time, spent the nights with them. Five adult pet dogs of various breeds were also present during the hand-raising with whom all pups established close relationships and readily submitted to them until the end of this study. This raising procedure of the Wolf Science Center () was adopted so that all study animals would be similarly socialized both with humans and conspecifics in order to allow us to make comparisons between the intra- and inter-specific interactions of dogs and wolves. At 5 months all pups were moved to 4000–8000 m^2^ enclosures. The enclosures were equipped with trees, bushes, logs, and shelters. Water for drinking was permanently available. The dogs and wolves received a diet of meat, milk products, and dry food throughout the study period. During the first months of their lives, they were fed several times per day, which was slowly reduced to being fed major meals once a day (dogs) and once every 3–4 days (wolves) according to their natural rhythm. Starting at the age of 4–5 months, no humans were continuously present in the enclosures, but all animals received intensive obedience training including sit, down, roll-over and leash walking on a daily basis. This training assured that the dogs and wolves were cooperative and attentive toward humans, and also allows veterinary checks without sedating the animals. Moreover, all animals participated in various behavioral tests every week, where they were also rewarded with food. All dogs and wolves were worked separately from their pack members on a daily basis. Participation in all training and testing sessions was voluntary.

**Table 1:** List of animals, indicating genetic relationships (litter), sex (male/female), age, and origin.

	Name	Sex	Litter	Born	Breeding facility
Wolf	Aragorn	M	1	2008	Herberstein, Austria
Wolf	Shima	F	1	2008	Herberstein, Austria
Wolf	Kaspar	M	2	2008	Herberstein, Austria
Wolf	Tatonga	F	4	2009	Tripple D Farm, USA
Wolf	Nanuk	M	3	2009	Tripple D Farm, USA
Wolf	Geronimo	M	5	2009	Tripple D Farm, USA
Wolf	Yukon	F	5	2009	Tripple D Farm, USA
Wolf	Cherokee	M	6	2009	Zoo Basel
Wolf	Apache	M	6	2009	Zoo Basel
Wolf	Kenai	M	7	2010	Parc Safari, Canada
Wolf	Wapi	M	7	2010	Parc Safari, Canada
Dog	Rafiki	M	1	2009	Tengelic; Hungary
Dog	Alika	F	1	2009	Tengelic; Hungary
Dog	Kilio	M	2	2009	Paks, Hungary
Dog	Maisha	M	2	2009	Paks, Hungary
Dog	Asali	M	3	2010	Siofok, Hungary
Dog	Binti	F	3	2010	Siofok, Hungary
Dog	Bashira	F	4	2010	Paks, Hungary
Dog	Hakima	M	4	2010	Paks, Hungary
Dog	Meru	M	5	2010	Velence, Hungary
Dog	Nuru	M	6	2011	Paks, Hungary
Dog	Zuri	F	6	2011	Paks, Hungary
Dog	Layla	F	7	2011	Györ, Hungary
Dog	Bora	F	7	2011	Györ, Hungary
Dog	Nia	F	8	2011	Paks, Hungary

### EXPERIMENTAL SET-UP

**FIGURE 1:**
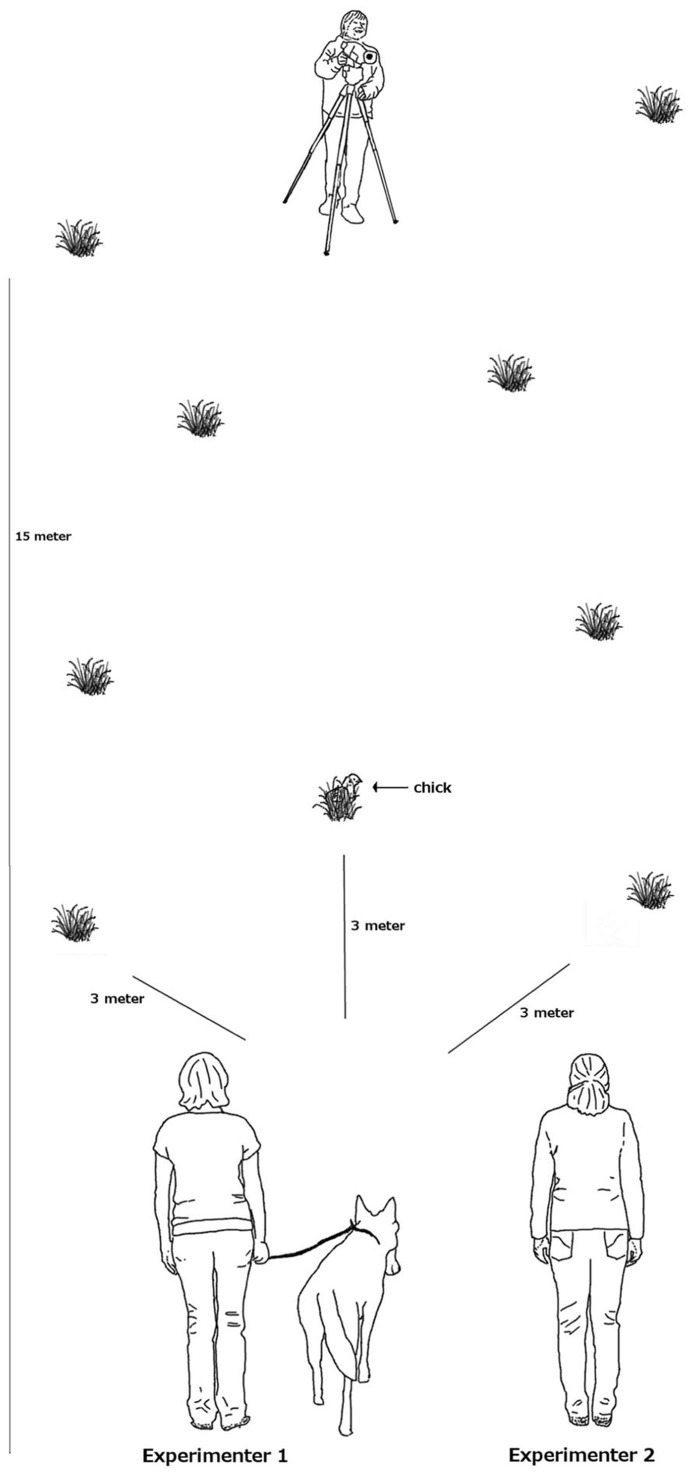
**Experimental set-up**.

To test whether and in what detail wolves and dogs pay attention to a human or dog demonstrator, we used a simple social learning task where the demonstrator placed a treat in one of three locations in a meadow. As treats we used dead, 1-day old chicks. Such chicks we use regularly as a reward in experiments because the animals are highly motivated to obtain them. The three locations were equidistant from the starting position of the subject at a distance of 3 m, either to the right, in the front or to the left of the starting position (see **Figure 1** for experimental set-up).

### EXPERIMENTAL CONDITIONS

All tests were conducted in different meadows (15 × 15 m) familiar to the animals from previous walks. A hand-raiser, the subject, two experimenters (E1, E2) and – depending on the demonstration – a pet dog were present during the experiments. Using a within-subject design, we tested every animal in all of the following five experimental conditions at each age of 4, 5, and 7 months:

DOG_DEM = Dog demonstration

DOG_CON = Dog control demonstration (no chick)

HUMAN_DEM = Human demonstration

HUMAN_CON = Human control demonstration (no chick)

SMELL_CON = Smell control (no demonstration, chick placed previously)

One experimental trial per condition was conducted per age. The five trials at each age were carried out over a time period of 2 weeks with the conditions blocked according to the type of demonstrator: e.g., in 1 week both human conditions were tested and in the other week both dog conditions. The smell control was randomly intermixed between those other trials. The sequence of all trials was pseudo-randomized between subjects as well as within subjects (across the various ages). Dogs and wolves were tested with similar sequences. We conducted a maximum of three experimental trials per day; tests with the same demonstrator (human/dog) were no more than 2 days apart. Each experimental trial was tested in a different meadow toward a different location (left, middle, right); the three meadows as well as the locations were randomly assigned across experimental conditions, between and within subjects with the restriction that neither the same meadow nor the same location was used in subsequent tests of the same subject or across subjects.

### PROCEDURE

Before each experimental trial, in the absence of the subject, the demonstrator (in case of the demonstrator dog after being sent with a hand signal to do so) walked twice along the direct line from the starting position to each of the three hiding locations to control for odor. Once the demonstrator had returned to the starting position, experimenter 1 approached with the subject on a 10-m leash from the other side of the starting position. During this approach and at the starting position, the subject was kept on a short leash and prevented from exploring. At the same time, experimenter 2 took position with a tripod and a camera 15 m across from the starting position on the other side of the meadow. After everybody was in position, the subjects received one of the 5 experimental conditions:

#### Dog demonstration (DOG_DEM)

The hand-raiser showed the chick to the subject and then gave it to the demonstrator dog that took it into the mouth in full view of the subject. While the subject was watching, the hand-raiser sent the demonstrator dog to the pre-assigned location, where it dropped the chick and returned to the starting position. During the demonstration, experimenter 1 held the subject short on the leash (approx. 1 m, rest of the leash was rolled up in the hand of the experimenter), but otherwise refrained from interacting with the animal. As soon as the demonstrator returned to the starting position, experimenter 1 dropped the rolled-up part of the 10-m leash, keeping only the end in the hand and thus released the subject. The subject was now free to do what it wanted within the 10-m radius of the leash. The hand-raiser, dog and experimenters refrained from interacting with the subject. The trial was terminated when the subject found the chick or after 2 min. During the entire time, experimenter 1 had a second chick in her pocket to control for odor in comparison with the Control and Smell Control trials when experimenter 1 had a chick in her pocket as well (see procedure below).

#### Dog control demonstration (DOG_CON)

The hand-raiser showed the chick to the subject and then gave it to experimenter 1 in full view of the subject. Experimenter 1 demonstratively put the chick into her pocket and then showed her empty hands to the subject (a sign that no treat will be given). While the subject was attending to the dog demonstrator, the hand-raiser sent the demonstrator dog (without a chick) to the pre-assigned location, where it then turned around and returned to the starting position. Otherwise, the procedure was identical to the Dog Demonstration. The trial was terminated if the subject went to the location where the demonstrator dog had turned or after 2 minutes.

#### Human demonstration (HUMAN_DEM)

The Human Demonstration was identical to the Dog Demonstration with the exception that no demonstrator dog was present and the hand-raiser, after taking the chick out from her pocket and showing it to the subject, carried the chick in her hand and bent down at the pre-assigned location to hide the chick. Then she returned to the starting position, showed her empty hands to the subject who was then released.

#### Human control demonstration (HUMAN_CON)

The Human Control Demonstration was identical to the Dog Control Demonstration with the exception that no demonstrator dog was present and the hand-raiser, after handing the chick over to experimenter 1, walked to and bent down at the pre-assigned location.

#### Smell control (SMELL_CON)

Before the subject was led to the starting position, the hand-raiser hid a chick at the pre-assigned location. After experimenter 1 and the subject arrived at the starting position, the hand-raiser showed another chick to the subject and then gave it to the experimenter 1 in full view of the subject identically to the human and dog control conditions. Experimenter 1 demonstratively put the chick into her pocket and then showed her empty hands to the subject. Afterward experimenter 1 dropped the rolled-up part of the 10-m leash, keeping only the end in the hand and thus releasing the subject. The subject was now free to do what it wanted within the 10-m radius of the leash. The hand-raiser and experimenter 1 refrained from interacting with the subject. The trial was terminated when the subject found the chick or after 2 min.

### DATA ANALYZES

From the videos, we extracted whether or not the subject found the chick (test and smell control conditions) or went to the point where the demonstrator turned back in the control conditions (endpoint). Moreover, we coded the latency to find the chick in the test condition or to check the endpoint in the control conditions. The start was defined as the first movement of the subject from experimenter 1 and the demonstrator/hand-raiser. Finally, we coded how long the animals watched the demonstration and once the demonstrator had returned to the starting position whether or not the animals looked at experimenter 1, who, in all trials, had a chick in her pocket.

Test videos were analyzed by Teresa Schmidjell, who was blind to the goal of the experiment. To confirm scoring consistency 20% of the videos were coded by a second independent coder. Spearman’s rank correlations (rho) were in general high: Test duration: 0.9; Endpoint or chick found yes/no: 0.85; Latency endpoint or chick found: 0.88; Latency leave: 0.89; Duration of demonstration: 0.91; Duration of looking at the demonstration: 0.94; Looking at the experimenter 1 yes/no: 0.81; Duration of looking at experimenter 1: 0.89.

When comparing the two test conditions (DOG_DEM, HUMAN_DEM) with the smell control (SMELL_CON) condition, or respectively the test conditions (DOG_DEM, HUMAN_DEM) with the control conditions (DOG_CON, HUMAN_CON), we calculated mixed effect models including the individual’s identity and the test trial as random factors. We analyzed whether the subjects’ success (defined as finding the chick) and latency to find the chick or investigating the end point was influenced by age, sex, condition, species, relative time sniffing at the ground, and relative observation time (comparison tests vs. control conditions). A binomial distribution was used to analyze the subjects’ success, whereas the latency to find the chick or investigate the endpoint was analyzed with a linear mixed effect model using a 1/√y transformation.

Furthermore, we analyzed whether age, sex, condition, and species had an influence on whether an individual was looking at the experimenter 1 or on the relative time the individuals spent observing the demonstration. A binomial distribution was used for whether an individual was looking at the experimenter 1. The analyzes were performed using the program R 2.11.1. (R Core Team 2010).

## RESULTS

### THE EFFECT OF A DEMONSTRATION

**FIGURE 2:**
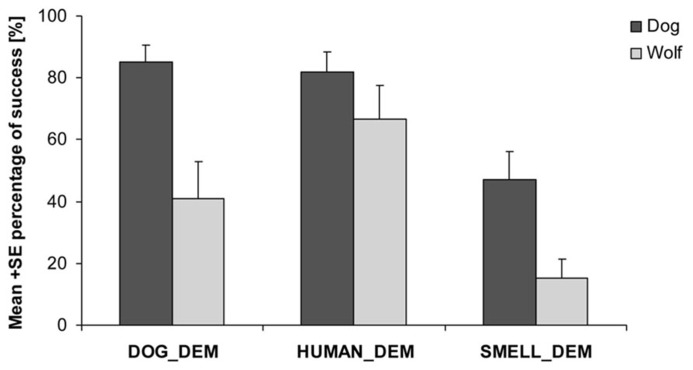
**Proportion of success finding the chick for wolves and dogs in the dog demonstrator (DOG_DEM), human demonstrator (HUMAN_DEM) and smell control (SMELL_CON) condition**.

We found that both dogs and wolves were more likely to find the chick after observing a demonstrator than if no demonstration had been provided (wolves: nlme: HUMAN_DEM vs. SMELL_CON: *t*_57_= 4.084, *p* < 0.001; DOG_DEM vs. SMELL_CON: *t*_57_= 2.642, *p* = 0.011; dogs: nlme: HUMAN_DEM vs. SMELL_CON: *t*_82_= 4.410, *p* < 0.001; DOG_DEM vs. SMELL_CON: *t*_82_= 4.151, *p* < 0.001). However, dogs were more likely to find the chick than wolves in all trials (nlme: *F*_1,29_= 20.120, *p* < 0.001, **Figure 2**) independent of whether the demonstrator was a dog or a human or whether there was a demonstration or not (nlme: species*DOG_DEM vs. species*HUMAN_DEM: *t*_135_= 1.46, *p* = 0.15; species*DOG_DEM vs. species*SMELL_CON: *t*_135_= 1.23, *p* = 0.22) suggesting that either dogs were more motivated to search for the chick or that, at least partly, they were more successful in using their sense of smell to find the chick.

**FIGURE 3:**
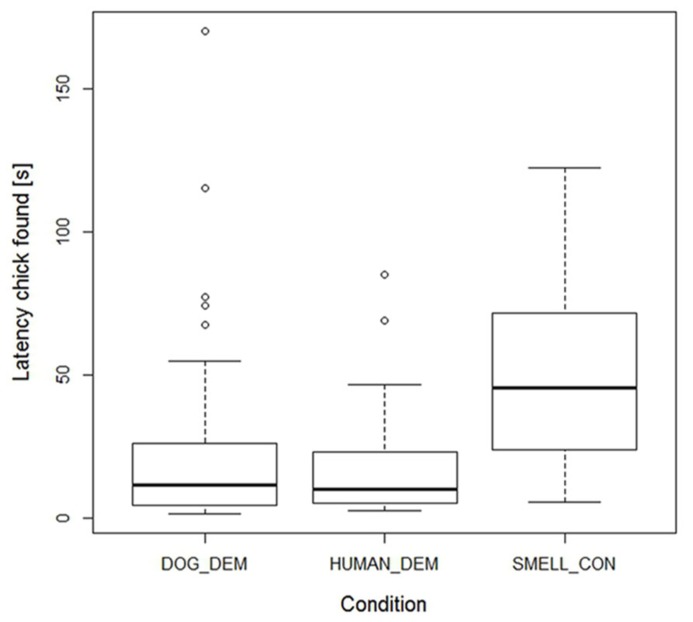
**Box plots showing the successful wolves and dogs’ latency to find the chick in the 3 conditions.** Boxes represent the interquartile range, bars within shaded boxes are median values, whiskers indicate the 5th and 95th percentiles, and open circles are outliers.

Motivation of dogs and wolves, however, seemed to be similar, since we found no difference in the latency to find a chick between successful wolves and dogs (lme: species: *F*_1,26_= 0.81, *p* = 0.38). Latency to find the chick was not influenced by age or sex either (lme: age: *F*_1,13_= 0.84, *p* = 0.38; sex: *F*_1,22_= 1.13, *p* = 0.30). Condition had a significant effect though (lme: *F*_2,123_= 14.951, *p* < 0.001), with animals needing more time in the smell control to find the chick than in the dog demonstration trials, but there was no difference between dog and human demonstration trials (lme: DOG_DEM vs. SMELL_CON: *t*_58_= -3.836, *p* < 0.001; DOG_DEM vs. HUMAN_DEM: *t*_58_= 0.15, *p* = 0.88; see **Figure 3**).

**FIGURE 4:**
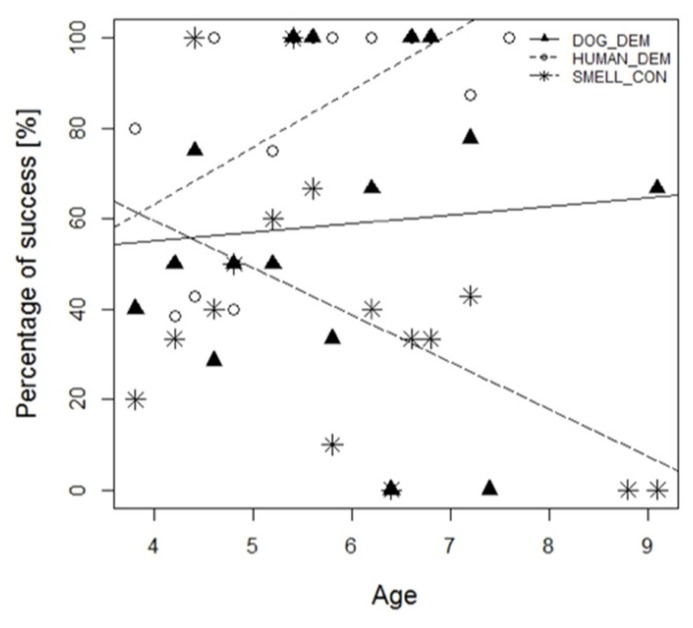
**The graph presents the percentage of success of all animals (wolves and dogs combined) at a certain age in the human demonstration (HUMAN_DEM), dog demonstration (DOG_DEM) and smell control (SMELL_CON) condition**.

Furthermore, in regard to the success of the animals we found an interaction between age and condition indicating that the likelihood to find a chick relative to the age of the individuals’ (dogs as well as wolves) differed between smell control and dog demonstration but not between human demonstration and dog demonstration (nlme: age*DOG_DEM vs. age*HUMAN_DEM: *t*_137_= 1.60, *p* = 0.11; age*DOG_DEM vs. age*SMELL_CON: *t*_137_= -2.405, *p* = 0.018). While there was an increase in success with increasing age in the human demonstration and dog demonstration test, success in the smell control decreased with increasing age suggesting that the animals learned to pay more attention to visual cues rather than relying on their sense of smell (**Figure 4**).

### DO THE ANIMALS PAY ATTENTION TO THE DETAILS OF THE DEMONSTRATION, e.g., DO THEY RECOGNIZE THAT THE CHICK IS NOT BEING HIDDEN IN THE DOG AND HUMAN CONTROL DEMONSTRATION TRIALS?

**FIGURE 5:**
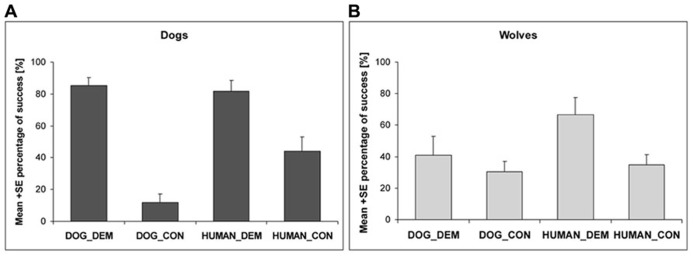
**The success of dogs (A) and wolves (B) in the dog and human demonstration (DOG_DEM and HUMAN_DEM) and control conditions (DOG_CON and HUMAN_CON)**.

When analyzing the likelihood to find the chicks or the endpoint, respectively, in the trials with a chick demonstration and a control demonstration, we found a significant interaction between species and dog demonstration trials (nlme: species*DOG_DEM vs. species*DOG_CON: *t*_212_= -4.418, *p* < 0.001) but not between species and human demonstrator trials (nlme: species*HUMAN_DEM vs. species*HUMAN_CON: *t*_212_= -0.76, *p* = 0.45). While dogs found the chick equally often after a human or dog demonstration (nlme: *t*_120_= 0.13, *p* = 0.90), they went less often to the endpoint during the human control than during the human demonstration (nlme: *t*_120_= -4.270, *p* < 0.001), and less often to the end point during the dog control compared to the dog demonstration condition (nlme: *t*_120_= 6.729, *p* < 0.001; **Figure 5A**). Wolves, however, found the chick more often after human than after dog demonstration (nlme: *t*_97_= 2.101, *p* = 0.038) and did not differentiate between the two different dog demonstrations (nlme: *t*_97_= -1.23, *p* = 0.22). Interestingly though, if the human demonstrated, they went to the endpoint fewer times during the human control compared to the human demonstration (nlme: *t*_97_= -2.731, *p* = 0.008; **Figure 5B**).

**FIGURE 6:**
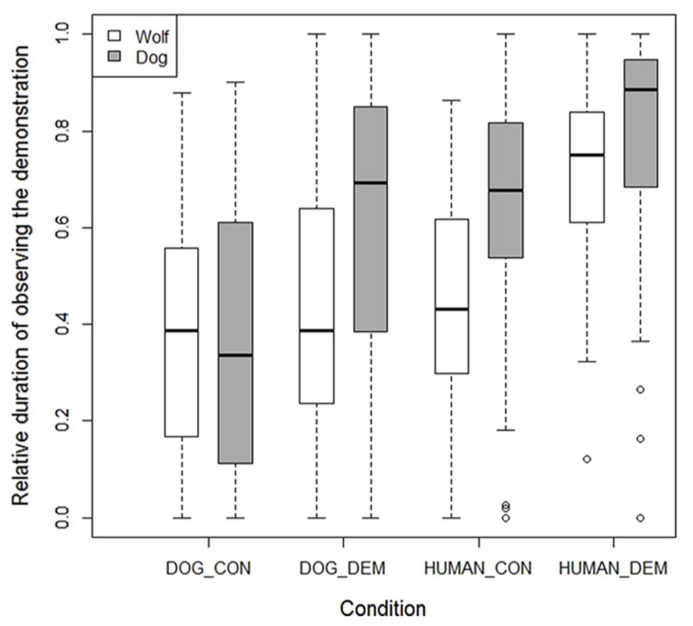
**Box plots showing the relative duration that the wolves and dogs observed the various demonstrations.** Boxes represent the interquartile range, bars within shaded boxes are median values, whiskers indicate the 5th and 95th percentiles, and open circles are outliers.

The surprising finding that the wolves were rather unsuccessful in finding the chick in the dog demonstration trials was partly explained by their lower interest in this demonstration compared to the dogs (lme: *F*_1,26_= 5.279, *p* = 0.030), while dogs and wolves watched the dog control demonstration for similar lengths of time (lme: *F*_1,26_= 0.15, *p* = 0.700). The dogs and wolves also paid similar attention to the human demonstration (lme: *F*_1,26_= 0.23, *p* = 0.630), but here dogs actually paid more attention to the human control demonstration than the wolves (lme: *F*_1,26_= 13.540, *p* = 0.001; see **Figure 6**).

## DISCUSSION

Overall, we found that wolves and dogs benefitted from a demonstration independent of whether a conspecific or human had provided the information. Regarding how much attention they paid to the details of the demonstration, we found no difference between wolves and dogs when a human was the demonstrator: both groups clearly differentiated whether or not the human demonstrator actually hid a chick or only pretended to do so. However, while the dogs showed the same behavior after a conspecific demonstration, the wolves paid less attention to the dog demonstration and control trials than the dogs and did not differentiate between them.

The result that both wolves and dogs showed a clear benefit from the demonstration suggests that they primarily relied on the visual information provided by the demonstrator rather than olfactory cues. Although it is clear that canines have an extraordinary sense of smell, little is known regarding in what situations they actually use olfactory cues if not specifically trained to do so. Thus, for example, while the popular consensus is that olfaction is very important for hunting (Asa and Mech, 1995), two studies that experimentally investigated the role of olfactory, auditory, and visual cues found that visual cues are the most important ones for hunting in red foxes (Österholm, 1964) and coyotes (Wells, 1978; Wells and Lehner, 1978). Also another study using a two-choice pointing task showed that dogs did not use their nose to find the hidden food, but instead went to the empty container if the experimenter had pointed at that (Szetei et al., 2003). These results might suggest that in canines visual information may easily override odor cues if searching for hidden food items (but see Gazit and Terkel, 2003 for a contrary example in explosives detection trained dogs).

However, two aspects hint toward a role of the animals’ sense of smell in our experiment: (1) all animals sometimes found the chick in the smell control trials when no visual information was provided, e.g., they most likely used their sense of smell to find the hidden food and (2) dogs clearly outperformed the wolves both in demonstration as well as control trials. The reason for this latter difference is open for speculation. Differential motivation to search for the food, which could be caused for example by a different hunger status of the dogs and wolves or their different preferences for the chicks, would be one possible explanation, but the lack of a difference in the latency to find the chick between the two groups discounts that possibility. Alternatively, the dogs have a better sense of smell than the wolves to locate the hidden chicks. However, to our knowledge there is no experimental data which would support this idea. Another interesting finding is that both wolves and dogs relied less on their sense of smell as they grew older, relying more on the visual information provided by the demonstrators, which was reflected in the fact that they watched the demonstrations longer as they grew older and were less successful in the smell control trials.

Interestingly, the wolves used the human demonstration even more successfully than the dog demonstration suggesting that they, similarly to the dogs, accepted humans as a social partner. This can be explained by the intensive socialization of our animals at the Wolf Science Center. Alternatively, it is also conceivable that the animals did not regard the demonstration in terms of the social information provided, but extracted mainly non-social information about the food location (see also Mersmann et al., 2011 for a similar explanation).

When further analyzing our human and dog demonstrations, however, it became apparent that the species of the demonstrator played a major role in determining how close the dogs and the wolves paid attention to the details of the demonstration, suggesting that the social aspect is important. Both dogs and wolves differentiated between the human demonstration and human control condition, visiting the end point less often when no chick was actually hidden. While wolves and dogs observed the human demonstration trials to the same extent, the dogs paid more attention to the human control trials than the wolves did.

This finding is in line with the prediction of the domestication hypotheses which expects more a priori interest in humans in dogs than in wolves. Alternatively, wolves showed less interest in the control demonstration trials because they have a better causal understanding than the dogs and thus are bothered by the apparent lack of goal of the without-food demonstration (see also Topál et al., 2009). This latter argument would support the “*information processing hypothesis,”* which predicts that – due to the buffering effect of humans leading to a relaxation of natural selection on the problem-solving abilities of dogs–wolves have a better causal understanding than dogs (Frank, 1980) but see (Fiset and Plourde, 2012; Range et al., 2012b).

In contrast to the wolves, the dogs also differentiated between the demonstration and the control trials after the demonstration of a conspecific. Interestingly, the wolves were less interested in the dog demonstration than the dogs even when the demonstrator had a chick in its mouth, whereas both groups paid similar attention to the demonstration in the dog control trials. This result is in contrast with our expectations based on the domestication hypotheses, especially because it was not the case that the wolves investigated the end point less often in the human control condition compared to the other three conditions, but rather the wolves went to the end point more often in the human demonstration trials than in any of the other three conditions. This suggests that they paid special attention to the human demonstration also when compared to the dog demonstration. Several explanations could account for this result:

First, the demonstrator dogs might not have been as important for the wolves as for the dogs since they are not conspecifics in the strict sense. For example, it has been shown in different monkey species, that monkeys devote more attention to conspecifics than to individuals from a closely related species (rhesus vs. Japanese macaques) despite significant physical and behavioral similarities (Fujita, 1993). However, in our case, this explanation is unlikely, since all the wolves had a close social relationship with the demonstrator dogs, who had been part of the hand-raising of the subjects from the beginning on. They greeted, played with and readily submitted to them, i.e., accepted the dogs as dominant pack-mates. More importantly, in other experiments the wolves readily followed the information provided by the same demonstrator dogs and in several experiments outperformed the pack dogs, suggesting that they do pay close attention to these pet dogs (Range and Viranyi, 2011; Range and Viranyi, submitted; Viranyi and Range, submitted).

Second, wolves accepted the ownership of the chick by the demonstrator dog and thus did not want to challenge the demonstrator by looking for the chick as long as the dog was present. However, also this is in contrast with our observations that the wolves challenge dominant conspecifics for food much more than dogs (Range et al., in preparation; Viranyi et al., in preparation).

Third, our wolves have a very cooperative relationship with their human hand raisers, who functioned as demonstrators. We train the animals at the Wolf Science Center on a daily basis trying to avoid any conflicts between the animals and the humans. Thus, it is possible that the wolves were more interested in the humans who usually reward them with food during training sessions. The pet dogs, on the other hand, are just not as interesting since the wolves do not expect them to provide them with food especially since the reward (chick) was not of a size that a wolf would usually cache. This behavior again could be based on a better causal understanding of wolves compared to dogs.

Finally, it is possible that the wolves focused on different details of the demonstration than the dogs. The demonstrator dogs did not like to take the dead chicks in their mouths and clearly showed their resistance by turning their head or trying to spit the chick out. In monkeys, it has been shown that at least one species is sensitive to a display of disgust, adjusting their behavior accordingly (Snowdon and Boe, 2003). If wolves are similarly sensitive, they might have discarded the chicks as something inedible. Our result that the wolves paid less attention than the dogs to the dog demonstrator when it had a chick in its mouth, but not in the control condition when it did not carry a chick, seems to be in line with this explanation.

These latter two speculations suggest that wolves and dogs may differ in regard to how attentive they are toward behaviors of their social partners. Being more attentive to the behavioral details of their social partner’s actions is probably more important for wolves than dogs. Wolves are cooperative breeders (Mech, 1970; Mech and Boitani, 2003) relying on close action coordination with pack members when defending their territories and hunting large game (Mech, 1970; Mech and Boitani, 2003). Dogs, however, though closely related phylogenetically (Scott and Fuller, 1965; Savolainen et al., 2002; Pang et al., 2009), differ fundamentally not just in regard to their closeness to humans, but also in their breeding system and possibly other intraspecific interactions (Boitani and Ciucci, 1995; Butler et al., 2004); but see (Bonanni et al., 2010b). Although feral dogs live in pack-like social groups (Bonanni et al., 2010a; Cafazzo et al., 2010), female feral dogs raise their pups alone (Daniels and Bekoff, 1989; Boitani and Ciucci, 1995) or with the help of the fathers that in some populations may contribute to the defense of the pups but rarely feed them (Pal, 2005). Consequently, the overall higher dependency on cooperative interactions with conspecifics that require close action coordination in order to be successful [i.e., *canine cooperation hypothesis *(Range et al., 2012a)], might explain why our wolves behaved differently toward the human and dog demonstrator who differ quite extensively in regard to their roles when interacting with our study animals.

Overall, these results suggest that the wolves and dogs do not differ very much from each other in regard to their ability to use information provided by a human or conspecific demonstrator in a local enhancement test. The minor differences in performance can be explained by (1) higher propensity of wolves to pay attention to the details and/or (2) a greater tendency of dogs to concentrate on what humans do, not matter whether this is causally justified or not.

## Conflict of Interest Statement

The authors declare that the research was conducted in the absence of any commercial or financial relationships that could be construed as a potential conflict of interest.
